# Prognostic Value of Thyroid Hormone FT3 in General Patients Admitted to the Intensive Care Unit

**DOI:** 10.1155/2020/6329548

**Published:** 2020-07-12

**Authors:** Jianying Guo, Yanyan Hong, Zhiyong Wang, Yukun Li

**Affiliations:** ^1^Department of Endocrinology, The Third Hospital of Hebei Medical University, Shijiazhuang 050051, China; ^2^Department of Critical Care Medicine, The Third Hospital of Hebei Medical University, Shijiazhuang 050051, China; ^3^Infirmary of Shijiazhuang Institute of Technology, Shijiazhuang 050228, China

## Abstract

Low plasma triiodothyronine (T3) concentration indicates nonthyroidal illness syndrome (NTIS), which might be associated with a poor outcome in patients in the intensive care unit (ICU). This study evaluated the relationship between NTIS and prognostic indicators in patients admitted to the ICU and examined the fT3 cut-off points that could be associated with 28-day mortality. This prospective observational study included patients admitted to the ICU of The Third Hospital of Hebei Medical University from February to November 2018. The baseline variables and the occurrence of low free T3 (FT3) were collected. The patients were divided into the NTIS (FT3 < 3.28) and non-NTIS groups. Among 305 patients, 118 (38.7%) were in the NTIS group. FT3 (*P* < 0.001) and FT4 (*P* = 0.001) were lower, while the 28-day mortality rate (*P* < 0.001) and hospitalization expenses in ICU (*P* = 0.001) were higher in the NTIS group. The univariable analyses identified NTIS, FT3, free thyroxine/FT3, APACHEII, sequential organ failure score, duration of mechanical ventilation, creatinine, oxygenation index, white blood cells, albumin, age, and brain natriuretic peptide as being associated with 28-day mortality (all *P* < 0.05). The cut-off value of FT3 for 28-day mortality was 2.88 pmol/L. The 28-day mortality rate and hospitalization expenses in the ICU were higher in patients with NTIS. NTIS was independently associated with 28-day mortality.

## 1. Background

Patients with normal basic thyroid function often present with abnormal thyroid hormone levels because of trauma, infection, surgery, inflammation, and other factors. This commonly results in decreased plasma triiodothyronine (T3), decreased or normal thyroxine (T4) and thyroid-stimulating hormone (TSH), and increased plasma reverse T3 (rT3) [1]. These alterations in thyroid hormones are known as the nonthyroidal illness syndrome (NTIS), euthyroid sick syndrome, or low T3 syndrome [2]. The magnitude of the changes in serum T3 and rT3 reflects the severity of the illness; patients with mild to moderate NTIS usually have normal plasma T4 and TSH concentrations, whereas patients with a more severe or prolonged illness will often have low serum T4 and TSH [3].

Some authors consider NTIS not to be a disease by itself but rather a manifestation of a self-protection mechanism in critically ill patients [4]. This syndrome is possibly associated with decreased 5′-deiodinase activity and increased 5-deiodination that results in more inactivation of thyroid hormone and the production of 3T3 caused by various factors activated by a systemic inflammatory reaction, decreasing the metabolism of T4 into active T3 [5, 6], but the mechanism remains unclear [3]. Studies have shown that NTIS is associated with an adverse prognosis in acute myocardial infarction [7], heart failure [8], sepsis [9], severe trauma [10], and acute respiratory distress syndrome [11]. T3 or free T3 (FT3) can be considered a prognostic indicator in myocardial infarction and heart failure [7, 8]. It was also found that there was an association between the decrease in T3 or FT3 levels and mortality in intensive care unit (ICU) patients [9, 11–13].

A study of 480 ICU patients from China published in 2012 suggested that FT3 can be used as a predictor of all-cause mortality in ICU patients [14]. Moreover, the predictive ability was improved when combined with the APACHE II score [14]. Nevertheless, over half of the subjects had cardiovascular disease and cardiopulmonary failure, with a mean age of 72 years and overall mortality rate as high as 19.2%. Therefore, the results of that study could not be representative of most ICU patients in China and around the world. Subgroup analysis suggested that FT3 might only have a prognostic value in cardiac diseases and not sepsis or other major ICU diseases [14]. Other studies in smaller numbers of critically ill patients suggest that the FT3 levels might help predict mortality in a more general population of ICU patients [15, 16]. A systematic review of nine studies concluded that whether current thyroid hormone level could be used as a prognostic indicator of sepsis mortality needed further study [9].

Therefore, further research is needed to clarify the association between NTIS and the prognosis of patients in the ICU. In particular, the value of FT3 as a prognostic marker for mortality in the general ICU population needs further investigation. The aim of this study was to undertake a prospective investigation of patients admitted to the ICU to investigate the incidence of NTIS and the value of a decreased FT3 level as a prognostic marker of mortality. The study also examined the fT3 cut-off point that could be associated with 28-day mortality.

## 2. Material and Methods

### 2.1. Patients

This prospective observational study recruited patients admitted to the ICU of The Third Hospital of Hebei Medical University between February 2018 and November 2018. The inclusion criteria were (1) 16-80 years old, (2) patients with organ failure who required supportive treatments or intensive monitoring, and with an APPACHE II score > 10, (3) no history of abnormal thyroid function according to the patient's medical chart, and (4) no history of thyroid diseases such as hyperthyroidism, hypothyroidism, and thyroid tumors. The exclusion criteria were (1) patients with less than 24 h stay in the ICU, (2) patients who donate organs, (3) pregnant women or breastfeeding women, (4) patients with endocrine tumors, or (5) patients administered with antithyroid drugs or other iodine-containing drugs, e.g., amiodarone. The enrolled patients were divided into the NTIS group (FT3 < 3.28 pmol/L) and the non-NTIS group based on their FT3 levels measured soon after hospital admission.

The study was approved by the ethics committee of The Third Hospital of Hebei Medical University (No. 2018-019-1), and written informed consent was obtained.

### 2.2. Clinical Data Collection and Examination Methods

During the study period, 400 patients were admitted to the ICU, but 95 were not included because of the eligibility criteria. No patient withdrew from the study. A total of 305 patients were enrolled in the study. The cause of ICU admission included sepsis or sepsis shock, body injury, brain injury, severe disease, after operation, tumor, and pulmonary embolism. Body injury was defined as trauma to limbs and internal organs. Brain injury was defined as brain trauma and nerve damage. Severe disease included severe liver and kidney damage.

Levels of FT3, FT4, and TSH were measured in the enrolled patients within 24 h of admission. A radioimmunoassay was used for their detection in the Department of Nuclear Medicine of our hospital (Beckman DXI800, USA), with the normal reference values of 3.28-6.47 pmol/L for FT3, 7.64-16.3 pmol/L for FT4, and 0.49-4.91 mIU/L for TSH, respectively.

The vital signs and related laboratory indicators of patients were recorded, including albumin (ALB), brain natriuretic peptide (BNP), procalcitonin (PCT), white blood cells (WBC), hemoglobin (HGB), platelet (PLT), creatinine (CREA), total bilirubin (TBil), prothrombin time (PT), activated partial thromboplastin time (APTT), fibrinogen 9FIB), blood gas analysis, and lactic acid. Blood gas analysis and lactic acid levels were detected in the ICU, with arterial blood extracted in patients and tested using a multifunction blood gas analyzer (Rayto, USA). The remaining laboratory indexes were measured by the Laboratory Department of our hospital.

The enrolled patients were subjected to the acute physiology and chronic health evaluation II score (APACHE II), Glasgow coma scale (GCS), and sequential organ failure score (SOFA). All tests and scores were completed within 24 h of admission to the ICU. The duration of mechanical ventilation, length of stay in ICU, and costs of hospitalization were recorded.

### 2.3. Definitions and Follow-Up

The diagnostic criteria of NTIS was an FT3 level lower than the lowest normal value in our hospital, i.e., <3.28 pmol/L, with FT4 lower than or equal to the normal range of detection, and TSH in the normal range [1]. The patients were followed up for 28 days (in the hospital) to evaluate their survival outcomes and by phone when discharged.

### 2.4. Statistical Analysis

All data were analyzed using SPSS 19.0 (IBM Corp., USA). Continuous variables were tested for normal distribution using the Kolmogorov-Smirnov test. Continuous data were expressed as means ± standard deviation (SD) if they met the normal distribution, and the independent-sample *t*-test was used for intergroup comparison. Nonnormally distributed continuous data were expressed as medians (interquartile range (IQR)). The Mann–Whitney *U*-test was used for comparison. >Categorical data were expressed as percentages, and the chi-squared test was used for intergroup comparison. Univariable and multivariable logistic analyses were conducted. Receiver operating characteristic (ROC) curves were used to detect the cut-off value of FT3 for 28-day mortality. Variables with *P* < 0.05 were included in the multivariable logistic analysis (stepwise), and multicollinearity was considered. Bilateral *α* = 0.05 was used as the threshold to determine significance.

## 3. Results

### 3.1. Baseline of Patient Characteristics

There were 209 males and 96 females, with a mean age of 55.3 ± 15.7 years. There were 118 patients in the NTIS group, accounting for 38.7%, and 187 cases in the non-NTIS group. Details of the two groups are shown in Table 1.

There were differences between the NTIS and non-NTIS group in terms of age, diagnosis leading to ICU admission, APACHE II, SOFA, FT3, FT4, ALB, PCT, CREA, BNP, and PLT (all *P* < 0.05). The levels of FT3 and FT4 were lower in the NTIS group than those in the non-NTIS group (2.72 ± 0.40 pmol/L vs. 3.97 ± 0.63, *P* < 0.001; 11.79 ± 2.83 pmol/L vs. 13.59 ± 5.37, *P* = 0.001).

Compared with the survivors, the nonsurvivors were older, had a lower proportion of trauma, had higher APACHE II scores, had lower GCS scores, had higher SOFA scores, and had lower fT3 levels (all *P* < 0.05) (Table 2).

### 3.2. Comparison of Outcomes in the Two Groups

The 28-day mortality in the NTIS group was 19.5%, which was higher than that in the non-NTIS group (6.4%, *P* < 0.001). There was no obvious difference in the use of mechanical ventilation between the two groups. The length of stay in the ICU in the NTIS group was slightly longer than that in the non-NTIS group, but there was no significant difference between the groups (*P* = 0.094). The costs of hospitalization in ICU were higher in the NTIS group than in the non-NTIS group (*P* < 0.001) (Table 3).

### 3.3. Factors Associated with 28-Day Mortality

The univariable analyses suggested that the 28-day mortality of patients admitted to the ICU was associated with age, NTIS, FT3, FT4/FT3, APACHE II, SOFA, duration of mechanical ventilation, CREA, oxygenation index, WBC, ALB, and BNP (*P* < 0.05), as shown in Table 4.

Using multivariable logistical regression analysis of the 28-day mortality rate, after adjusting for duration of mechanical ventilation, CREA, oxygenation index, WBC, ALB, age, and BNP, NTIS was an independent factor for 28-day mortality (OR = 2.966, 95% CI: 1.68-7.41, *P* = 0.012) (Table 4).

### 3.4. ROC Curve Analysis of FT3 and 28-Day Mortality

**Figure 1:**
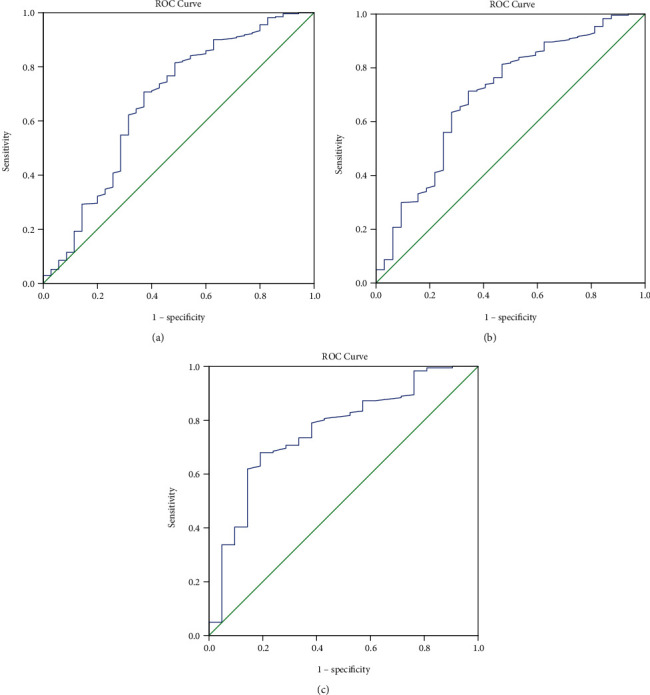
Receiver operating characteristic (ROC) curve for free triiodothyronine (FT3) level and 28-day mortality. The cut-off value of FT3 for 28-day mortality was 2.88 pmol/L, determined based on the Youden index. (a) ROC for all the patients, when cut-off of FT3 was 2.88 pmol/L, area under the curve (AUC) was 0.671 (95% CI: 0.563, 0.779), sensitivity was 0.514, and specificity was 0.815. (b) ROC for patients excluding those after an operation, when cut-off of FT3 was 2.875 pmol/L, AUC was 0.701 (95% CI: 0.598, 0.804), sensitivity was 0.504, and specificity was 0.813. (c) ROC for patients excluding those after an operation and those with brain injury, when cut-off of FT3 was 3.165 pmol/L, AUC was 0.76 (95% CI: 0.652, 0.869), sensitivity was 0.81, and specificity was 0.669.

The ROC curve analysis that included all patients suggested that a cut-off value of 2.88 pmol/L FT3 was related to 28-day mortality with an AUC of 0.671, sensitivity of 0.514, and specificity of 0.815. This suggests that patients who have a level of FT3 lower than 2.88 pmol/L are at high risk of death in the ICU (Figure 1 and Table 5). When the patients were reanalyzed after excluding those who underwent operations, a cut-off value of 2.875 pmol/L provided an AUC of 0.701 with sensitivity 0.504 and specificity 0.813. Further excluding patients with brain injury along with those who underwent operation provided an AUC of 0.76 with sensitivity 0.81 and specificity 0.669.

## 4. Discussion

This study investigated the relationship between NTIS and prognosis in patients admitted to the ICU. The results showed that 38.7% of the patients had NTIS in the ICU. There were differences between the NTIS and non-NTIS groups in terms of age, diagnosis leading to ICU admission, APACHE II, SOFA, FT3, FT4, ALB, PCT, CREA, BNP, and PLT. The 28-day mortality in the NTIS group was 19.5%, which was significantly higher than 6.4% in the non-NTIS group (*P* < 0.001). The costs of hospitalization in ICU were higher in the NTIS group than in the non-NTIS group (*P* < 0.001). The univariable analyses suggested that the 28-day mortality of patients admitted to the ICU was associated with age, NTIS, FT3, FT4/FT3, APACHE II, SOFA, duration of mechanical ventilation, CREA, oxygenation index, WBC, ALB, and BNP. The ROC analysis suggested that a cut-off value of 2.88 pmol/L FT3 indicated a higher risk of 28-day mortality. Excluding the patients who underwent operations or had brain injury further increased the AUC of the ROC analysis. Both the APACHE II and SOFA scores are complex clinical scoring systems consisting of large numbers of indicators that offer significant advantages over a single indicator, and no single indicator has been reported to date to exceed the APACHE II score in terms of the prognostic value for mortality [17]. Although FT3 has a lower diagnostic value than both scores, it has a specificity of 81.5% as a single indicator, which is still of great prognostic value. High FT3 specificity indicates low false-positive rates. In clinical practice, FT3 is a single laboratory test and might quickly and objectively provide information about the prognosis. FT3 results can be available within 2-4 h after admission. In contrast, APACHE II and SOFA scores take more time to be determined and can be subjectively influenced. They are impractical in ICUs with a heavy workload.

This study suggests that NTIS is an important indicator of 28-day mortality in patients in the ICU. The ROC analysis suggested that patients admitted to the ICU with an FT3 level of <2.88 pmol/L are at a high risk of death. Overall, these results agree with the study by Wang et al. [14]. Nevertheless, there are differences in disease types and patient age between the two studies. The previous study had a high rate of patients over 71 years with cardiovascular diseases and cardiorespiratory function failure [12], while the present study had a younger population with many cases of severe trauma. Thyroid hormones can increase the heart rate and myocardial contractility, improve diastolic cardiac function, and reduce systemic vascular resistance [18], having a significant effect on cardiac and circulatory function. A decreased thyroid level has a great influence on the function of the heart and circulation [19]. Changes in thyroid hormone levels are evident in myocardial infarction, heart failure, and other diseases and can thus be considered a predictor of multiple prognostic indicators [7, 8, 20]. Furthermore, other studies [21, 22] also suggested that the selective application of thyroid hormone in patients with myocardial infarction and extracorporeal circulation can improve the survival rates. The results of the present study suggest that NTIS might also be an important prognostic marker in patients with other diagnoses, not cardiac related, in the ICU. At the same time, it has been suggested that decreased FT3 is related to a low autometabolic rate. When the patients who had undergone operations were excluded from the ROC analysis, the AUC increased, suggesting that FT3 levels might be more useful in some patient populations than in others. The AUC increased further when the patients with brain injury were excluded along those who had undergone operations, further supporting the view that the specific diagnoses should be considered when using FT3.

In the present study, the cut-off point for predicting mortality was 2.88 pmol/L. Yu et al. [23] reported a cut-off of 3.685 pmol/L for mortality in patients with acute myocardial infarction. Su et al. [24] reported a cut-off point of 2.415 pmol/L for the prediction of cardiovascular death in the ICU. Different patient populations might influence the determination of the cut-off point, and large studies are needed to determine the exact values.

Consistent with other findings, the present study found that NTIS, defined as those with FT3 below the laboratory minimum normal value (FT3 < 3.28), indicated a worse prognosis than non-NTIS. The 28-day mortality of patients admitted to the ICU was much higher than in the non-NTIS patients and was associated with higher hospitalization costs. In a previous study with the duration of mechanical ventilation of NTIS patients as the main objective, published in Chest in 2009 [25], the results suggested that the duration of mechanical ventilation in patients with NTIS was significantly prolonged, but this was not found in this study. Furthermore, our study indicated that there was no significant difference in the use of mechanical ventilation between the two groups, which may be related to the incorporation of noninvasive ventilator-assisted ventilation into the scope of mechanical ventilation. Meanwhile, no significant difference was found, although the ICU duration in the NTIS group was longer than that in the non-NTIS group, which might be attributed to the lack of strict assurance concerning the indications for the discharge of the patients from the ICU. Specifically, many patients were discharged or had transfer delays out of the ICU owing to various reasons other than the condition of illness, whereas many patients who needed further treatments were transferred ahead of schedule due to the costs of treatment.

It should be highlighted that there were some limitations to this study. First, as a prospective predictive study, the sample size was still relatively small, without detailed stratified analysis, and with unbalanced types of diseases and a relatively high proportion of trauma patients, thereby affecting the estimation of overall mortality risk of patients admitted to the ICU. Multicenter studies should be designed in the future to ensure the diversity and balance of diseases. Second, a number of critically ill patients in the study chose to abandon or terminate treatment and voluntarily discharged themselves for various reasons such as economic considerations, which objectively increased the 28-day mortality rate, and had a certain degree of interference with the results. In addition, the 28-day mortality in patients admitted to the ICU is intimately associated with the intensity of treatment. Intensified ICU support therapy can make some patients survive 28 days, but the final prognosis is still poor. Therefore, the 90-day mortality rate should be further analyzed. Third, FT3, FT4, and TSH are routinely measured upon admission to the ICU, but thyroid-related antibody, thyrotropin receptor antibody, thyroglobulin, rT3, and thyroid B-ultrasound are not routinely done. Fourth, due to the wide variety of conditions being included and the different possible treatments for each condition, we could not include the treatments in the analyses. Fifth, the dynamic measurement of FT3 levels could represent more the actual condition of the patients and could detect more cases of NTIS, but the FT3 levels were not dynamically detected in this study. Finally, the total F3 levels were not measured.

## 5. Conclusion

In conclusion, NTIS was independently associated with a higher risk of 28-day mortality in patients admitted to the ICU. Although fT3 levels are not independently associated with ICU prognosis, the results suggest a cut-off value of fT3 levels that might be used for evaluating the prognosis of the patients. Future studies should examine the combination of fT3 levels with other biomarkers to construct a prognostic model for the ICU.

## Figures and Tables

**Table 1:** Baseline of characteristics of the study subjects.

Variable	Total (*n* = 305)	NTIS (*n* = 118)	Non-NTIS (*n* = 187)	*P*
Males (%)	31.5	30.5	32.1	0.96
Age (years)	55.3 ± 15.7	57.5 ± 16.6	54.0 ± 15.0	0.06
Principal diagnosis leading to ICU admission (%)				<0.001
Sepsis or sepsis shock	31	25	6	
Bodily injury	137	44	99	
Brain injury	71	18	53	
Severe disease of medicine	23	9	14	
After operation	32	18	14	
Tumor	9	7	2	
Pulmonary embolism	2	1	1	
APACHE II score	12.69 ± 8.06	15.44 ± 9.09	10.95 ± 6.80	<0.001
GCS score	12.2 ± 4.6	12.2 ± 4.5	12.2 ± 4.6	0.97
SOFA score	5.1 ± 3.9	6.3 ± 4.3	4.4 ± 3.4	<0.001
FT3 (pmol/L)	3.48 ± 0.82	2.72 ± 0.40	3.97 ± 0.63	<0.001
FT4 (pmol/L)	12.89 ± 4.64	11.79 ± 2.83	13.59 ± 5.37	0.001
TSH (mIU/L)	1.31 ± 3.35	1.67 ± 5.14	1.09 ± 1.27	0.148
Vital signs when enrolled				
*T* (°C)	35.93 ± 7.01	36.38 ± 5.97	35.66 ± 7.60	0.387
HR	90.9 ± 34.6	94.3 ± 37.0	88.8 ± 33.0	0.172
SBP (mmHg)	119.8 ± 40.2	115.6 ± 42.2	122.4 ± 38.7	0.149
DBP (mmHg)	65.6 ± 22.9	63.2 ± 24.4	67.2 ± 21.8	0.134
RR	21.4 ± 12.0	20.7 ± 8.8	21.8 ± 13.6	0.448
ALB (g/L)	28.29 ± 7.93	26.40 ± 6.93	29.48 ± 8.30	0.001
PCT (*μ*g/L)	2.6 ± 8.5	4.0 ± 11.2	1.7 ± 5.9	0.02
BNP (pg/mL)	103.8 ± 23.6	353.9 ± 978.9	115.2 ± 374.1	0.003
WBC (×10^9^/L)	12.0 ± 6.4	12.5 ± 7.56	11.6 ± 5.5	0.222
HGB (g/L)				
PLT (×10^9^/L)	146.5 ± 101.4	128.9 ± 105.3	157.5 ± 97.6	0.016
PT (s)	13.8 ± 8.2	14.8 ± 11.8	13.2 ± 4.7	0.101
APTT (s)	31.2 ± 14.2	33.4 ± 16.6	29.8 ± 12.3	0.032
FIB (g/L)	3.32 ± 2.00	3.35 ± 2.61	3.30 ± 1.50	0.848
Lactic acid (mmol/L)	2.78 ± 6.91	3.58 ± 10.46	2.26 ± 2.91	0.113
PCO_2_ (mmHg)	27.8 ± 17.7	26.7 ± 20.5	28.5 ± 15.7	0.379
Oxygenation index (PaO_2_/FIO_2_)	277.0 ± 140.4	270.6 ± 147.2	281.1 ± 136.2	0.535
CREA (*μ*mol/L)	65.7 ± 124.4	120.2 ± 165.8	80.2 ± 85.8	0.006
TBil (mmol/L)	29.0 ± 38.7	33.8 ± 51.0	25.9 ± 28.0	0.084
Na (mmol/L)	136.6 ± 22.3	136.0 ± 27.0	137.0 ± 18.9	0.744
K (mmol/L)	3.77 ± 0.92	3.74 ± 1.07	3.78 ± 0.81	0.664

NTIS: nonthyroidal illness syndrome; APACHE II score, acute physiology and chronic health evaluation II score; GCS: Glasgow coma scale; SOFA: sequential organ failure score; FT3: free triiodothyronine; FT4; free thyroxine; TSH: thyroid-stimulating hormone; *T*: temperature; HR: heart rate; SBP: systolic blood pressure; DBP: diastolic blood pressure; RR: respiratory rate; ALB: albumin; PCT: procalcitonin; BNP; brain natriuretic peptide; WBC: white blood cell; HGB: hemoglobin; PLT: blood platelet; PT: prothrombin time; APTT: activated partial thromboplastin time; FIB: fibrinogen; PCO_2_: pressure of carbon dioxide; PaO_2_: pressure of oxygen; FIO_2_: fraction of inspired oxygen; CREA: creatinine; TBil: total bilirubin.

**Table 2:** Baseline characteristics of the subjects according to 28-day mortality.

Characteristics	Survivors (*n* = 270)	Nonsurvivors (*n* = 35)	*P*
Males (%)	68.9	65.7	0.7
Ages (years)	54.3 ± 15.5	63.3 ± 15.0	0.001
Principal diagnosis leading to ICU admission (%)			<0.001
Sepsis or sepsis shock	8.6	25.7	
Severe trauma	50	5.7	
Brain injury or diseases	22.2	31.5	
Severe disease	6.3	17.1	
After operation	10.7	8.6	
Tumor	1.9	11.4	
Pulmonary embolism	0.7	0	
APACHE II score	11.19 ± 6.49	24.23 ± 9.60	<0.001
GCS score	12.81 ± 4.09	7.43 ± 5.21	<0.001
SOFA score	4.46 ± 3.14	10.00 ± 5.21	<0.001
FT3 (pmol/L)	2.77 ± 0.37	2.53 ± 0.47	0.009
FT4 (pmol/L)	11.95 ± 2.71	11.14 ± 3.25	0.22
TSH (mIU/L)	1.81 ± 5.70	1.06 ± 1.21	0.537
Vital signs when enrolled			
*T* (°C)	37.27 ± 0.84	37.36 ± 1.10	0.565
HR	93.1 ± 29.7	103.8 ± 34.7	0.052
SBP (mmHg)	127.56 ± 31.72	99.06 ± 35.41	<0.001
DBP (mmHg)	69.61 ± 18.37	56.38 ± 22.84	0.002
R	22.1 ± 9.9	23.4 ± 19.8	0.524
ALB (g/L)	29.04 ± 7.11	26.57 ± 6.66	0.049
PCT (*μ*g/L)	3.21 ± 8.51	9.77 ± 17.03	0.002
BNP(pg/mL)	439.1 ± 937.3	1193.0 ± 1301.4.4	0.005
WBC (×10^9^/L)	11.3 ± 5.1	17.0 ± 11.1	<0.001
HGB (g/L)	104.7 ± 21.7	99.9 ± 30.7	0.242
PLT (×10^9^/L)	151.4 ± 102.5	113.0 ± 84.7	0.035
PT (s)	13.7 ± 8.35	15.2 ± 6.94	0.329
APTT (s)	31.1 ± 13.2	32.9 ± 20.3	0.488
FIB (g/L)	3.35 ± 1.52	3.21 ± 4.15	0.697
Lac (mmol/L)	2.48 ± 6.91	5.08 ± 6.53	0.041
pH	7.32 ± 0.94	7.32 ± 0.17	0.937
BE (mmol/L)	−1.45 ± 4.62	−6.46 ± 8.45	<0.001
PCO_2_ (mmHg)	35.05 ± 10.50	36.56 ± 18.14	0.676
Oxygenation index (PaO_2_/FIO_2_)	287.3 ± 141.2	205.5 ± 104.8	0.001
CREA (*μ*mol/L)	81.9 ± 99.8	204.5 + 214.4	<0.001
TBil (mmol/L)	27.9 ± 35.2	40.3 ± 60.4	0.085
Na (mmol/L)	137.7 ± 16.1	139.7 ± 27.4	0.532
K (mmol/L)	3.81 ± 0.77	3.80 ± 1.28	0.931

APACHE II score: acute physiology and chronic health evaluation II score; GCS: Glasgow coma scale; SOFA; sequential organ failure score; fT3: free triiodothyronine; FT4: free thyroxine; TSH: thyroid-stimulating hormone; SBP: systolic blood pressure; DBP: diastolic blood pressure; PCT: procalcitonin; BNP: brain natriuretic peptide; HGB: hemoglobin; PLT: blood platelet; CREA: creatinine; TBil: total bilirubin; PT: prothrombin time; APTT: activated partial thromboplastin time; FIB; fibrinogen.

**Table 3:** Outcomes of the two groups.

Outcomes	NTIS (*n* = 118)	Non-NTIS (*n* = 187)	*P*
28-day mortality rate, *n* (%)	23 (19.5)	12 (6.4)	<0.001
MV rate, *n* (%)	92 (77.8)	140 (74.9)	0.78
Duration of MV (h), median (IQR)	56.5 (11.5, 117.6)	34 (7, 114)	0.097
Length of ICU stay (h), median (IQR)	109 (69.7, 219.5)	90 (58, 161)	0.094
Hospitalization expenses in ICU (×10,000 yuan), median (IQR)	4.57 (2.97, 9.00)	3.35 (2.27, 6.03)	0.001

MV: mechanical ventilation; ICU: intensive care unit; NTIS: nonthyroidal illness syndrome; IQR: interquartile range.

**Table 4:** Univariable and multivariable logistic regression analyses for 28d mortality.

	Univariable			Multivariable		
	OR	95% CI	*P* value	OR	95% CI	*P* value
NTIS	3.531	(1.68, 7.41)	0.001	2.966	(1.272, 6.915)	0.012
FT3	0.433	(0.26, 0.722)	0.001			
FT4	1.01	(0.941, 1.083)	0.789			
FT4/FT3	1.426	(1.121, 1.814)	0.004			
TSH	0.837	(0.585,1.197)	0.329			
APACHE II	1.231	(1.160, 1.308)	<0.001			
SOFA	1.378	(1.248, 1.520)	<0.001			
The duration of MV (h)	1.002	(1.000, 1.003)	0.046			
Lactic acid (mmol/L)	1.03	(0.991, 1.072)	0.137			
CREA (*μ*mol/L)	1.005	(1.002, 1.007)	<0.001			
Oxygenation index	0.995	(0.992, 0.998)	0.003	0.995	(0.991, 0.999)	0.007
WBC	1.121	(1.062, 1.183)	<0.001	1.118	(1.052, 1.187)	<0.001
ALB	0.962	(0.925, 1.000)	0.049	1.004	(1.002, 1.007)	0.001
Age (years)	1.045	(1.016, 1.075)	0.002			
BNP	1.001	(1.000, 1.001)	0.003			

Variables included in the multivariable logistic analysis were NTIS, the duration of MV, CREA, oxygenation index, WBC, ALB, and age. NTIS: nonthyroidal illness syndrome; FT3: free triiodothyronine; FT4: free thyroxine; TSH: thyroid-stimulating hormone; APACHE II score: acute physiology and chronic health evaluation II score; SOFA: sequential organ failure score; MV: mechanical ventilation; CREA: creatinine; WBC: white blood cell; ALB: albumin; BNP: brain natriuretic peptide.

**Table 5:** Receiver operating characteristic curve (ROC) of FT3 for 28-day mortality.

	AUC (95% CI)	Cut-off	Sensitivity	Specificity	*P* value
All patients	0.671 (0.563, 0.779)	2.88	0.815	0.514	<0.001
Patients excluding those after an operation	0.701 (0.598, 0.804)	2.875	0.813	0.504	<0.001
Patients excluding those after an operation and those with brain injury	0.76 (0.652, 0.869)	3.165	0.669	0.81	<0.001

## Data Availability

The datasets used and/or analyzed during the current study are available from the corresponding author on reasonable request.
